# ACE2-Mediated Infection of Immortalized Human Visceral Adipocytes by SARS-CoV-2

**DOI:** 10.3390/v17101311

**Published:** 2025-09-27

**Authors:** Francoise A. Gourronc, Megan I. Ahmann, Michael R. Rebagliati, Aloysius J. Klingelhutz

**Affiliations:** 1 Department of Microbiology and Immunology, University of Iowa, Iowa City, IA 52242, USA; francoise-gourronc@uiowa.edu (F.A.G.); michael-rebagliati@uiowa.edu (M.R.R.); 2 State Hygienic Laboratory, University of Iowa, Iowa City, IA 52241, USA; megan-ahmann@uiowa.edu; 3 Scientific Editing and Research Communication Core, University of Iowa, Iowa City, IA 52242, USA

**Keywords:** SARS-CoV-2, obesity, ACE2, COVID-19, visceral, fat, proinflammatory

## Abstract

Adipocytes can be infected by SARS-CoV-2, potentially contributing to the obesity-associated severity of COVID-19. Circumstantial evidence points to angiotensin-converting enzyme 2 (ACE2) as the necessary receptor for adipocyte infection, but this has not been demonstrated experimentally. Using differentiated immortalized human preadipocyte lines that we developed, we found that visceral adipocytes express higher levels of ACE2 and are more susceptible to SARS-CoV-2 spike (S)-mediated luciferase-VSV infection than subcutaneous adipocytes. Overexpression of ACE2 significantly increased infection, whereas knockout of ACE2 significantly decreased S-mediated infection. Visceral adipocytes at baseline were susceptible to infection by SARS-CoV-2 (Delta variant); however, increased levels of viral transcript with time were not apparent. ACE2 knockout significantly decreased the susceptibility of visceral adipocytes to SARS-CoV-2, whereas overexpression of ACE2 resulted in increased SARS-CoV-2 infection and was associated with increased viral transcript levels with time, as well as induction of IL6, a marker of the proinflammatory response. Our results demonstrate that ACE2 confers susceptibility to SARS-CoV-2 infection of visceral adipocytes. Higher levels of ACE2 in these cells may play a role in establishment of infection and a proinflammatory response, potentially leading to pathogenesis.

## 1. Introduction

Obesity is a significant comorbidity for COVID-19, but the precise reasons for this are still unclear [1,2]. Hypertrophic adipose tissue occurring during obesity is a common precursor to metabolic syndrome, making it a potentially significant player in pathogenesis caused by COVID-19 [3,4,5]. In particular, increased visceral adiposity, rather than subcutaneous adiposity, is associated with COVID-19 associated hospitalizations and mortality [1,2]. Adipose tissue secretes many different cytokines, chemokines, and hormones, collectively referred to as adipokines, some of which are specific for adipocytes, such as adiponectin, but others are not. When adipose tissue becomes dysfunctional—for example, when it accumulates excess lipids and turns hypertrophic—it becomes proinflammatory and exhibits alterations in hormone secretion [6,7]. Visceral adipose tissue is, in general, more metabolically active and becomes more inflammatory in obesity than subcutaneous adipose [8], which may partially explain why visceral adiposity leads to worse COVID outcomes.

The main cell types in adipose tissue are adipocytes, preadipocytes, adipose-derived stem cells, endothelial cells (in capillaries), and immune cells such as macrophages. How adipose tissue and, specifically, adipocytes, respond to viral infections has not been rigorously explored. Interestingly, in a recent paper, we demonstrated that Ebola Virus can infect adipocytes to induce a proinflammatory response [9]. Adipocytes express ACE2, the receptor for SARS-CoV-2, where it plays a role in the renin–angiotensin–aldosterone system (RAAS) [10]. As mentioned, increased visceral adipose tissue has been shown to strongly correlate with metabolic syndrome and COVID-19 severity [11,12]. Furthermore, visceral adipose tissue has higher transcript levels of *ACE2* than subcutaneous adipose in vivo [13]. Visceral adipocytes have also been shown to have higher levels of *ACE2* transcript than subcutaneous adipocytes [14].

SARS-CoV-2 RNA can be found in organs other than the lungs and nasopharyngeal tissue during the course of infection [15]. RNA has been detected in endothelial cells, the heart, kidney, and brain [16,17]. There have been many reports indicating the presence of SARS-CoV-2 RNA in the adipose tissue of humans [18,19]. Moreover, SARS-CoV-2 has been found to be present in the visceral and subcutaneous adipose tissue of hamsters infected with SARS-CoV-2, concomitant with a stronger proinflammatory response in visceral fat [20]. While it is unclear whether adipose is a site of productive infection in vivo, it has been shown that adipocytes are permissive for SARS-CoV-2 infection in vitro [20]. An additional study demonstrated efficient multi-cycle replication of SARS-CoV-2 in mature adipocytes, indicating both susceptibility and permissivity [18]. Furthermore, there is evidence that infection of adipose tissue by SARS-CoV-2 causes a proinflammatory response in macrophages and preadipocytes but a weak response in adipocytes themselves [21]. Interestingly, in this latter study, ACE2 expression could not be detected by western blot analysis in adipocytes or adipose tissue, leading the authors to conclude that virus entry into adipocytes is independent of ACE2. That visceral adipocytes have higher levels of ACE2 than subcutaneous adipocytes correlates well with SARS-CoV-2 susceptibility and suggests that ACE2 is the receptor [14,22]. However, it has not been demonstrated experimentally that this is the case. Here we leverage immortalized preadipocyte cell lines that we developed to confirm the role of ACE2 as a SARS-CoV-2 receptor in visceral adipocytes.

## 2. Materials and Methods

### 2.1. Cell Lines and Culture

The subcutaneous human cell line NPAD (Normal PreADipocyte) and clone (B) have been previously described [23,24]. For consistency and clarity in the current manuscript, we will refer to this cell line as SNPAD B to indicate its subcutaneous (S) origin. The visceral (V) NPAD lines VNPAD 2415 and VNPAD 30315 have also been described previously [25]. These cell lines were derived from primary preadipocytes acquired from consenting patient donors at the University of Iowa Hospitals and Clinics. The primary preadipocyte cells were immortalized using the reverse transcriptase component of telomerase (TERT) as well as HPV-16 E6 and E7 [23]. Additional unpublished subcutaneous and visceral preadipocyte cell lines were also derived by this methodology and are listed in Supplementary Table S1. All preadipocyte cell lines were cultured in Preadipocyte Growth Media (PGM2) (Lonza, Vacaville, CA, USA, PT-8002) without differentiation components, as previously described [26]. For differentiation, cells were plated at 100,000 cells/well in 12-well plates and allowed to become confluent, after which Preadipocyte Differentiation Media (PDM2) with differentiation components (Lonza, PT-8002) was added as described [26]. Cells were allowed to differentiate for 10 days before experiments were performed.

### 2.2. Transduction of Cells with ACE2

To over-express ACE2 in SNPADs and VNPADs, we transduced the cells as preadipocytes with a pLVX lentiviral vector that expresses codon-optimized human *ACE2* (made at Integrated DNA Technologies, Coralville, IA, USA) as well as a fluorescent mCherry reporter from an internal ribosome entry site (pLVX-ACE2-IRES-mCherry). To confirm transduction, cells were checked for mCherry fluorescence by microscopy and sorted at the Flow Cytometry Core based on red fluorescence within 72 h after transduction. mCherry-fluorescing cells were expanded, plated, and differentiated as above, and protein extracts were collected for western blot analysis, which was performed using an ACE2 antibody (R&D Systems, catalog number AF933) and a Beta-Actin antibody (Santa Cruz Biotechnology, Santa Cruz, CA, USA, catalog number SC-1616) as a control. Primary antibodies were detected using LI-COR secondary antibodies (LI-COR, Lincoln, NE, USA, LI-COR 926-68074 for ACE2 and LI-COR 926-54013 for Beta-Actin antibodies, respectively,) followed by imaging on a LI-COR Odyssey machine (Supplementary Figure S1).

### 2.3. Knockout of ACE2

Two different gRNAs targeting exon 1 of *ACE2* (gRNA1 and gRNA2) were separately cloned into lenti-CRISPR-v2 [27]. The gRNA sequences are listed in Supplementary Table S2. Pseudotyped replication-defective lentiviruses were generated as described [27]. VNPAD 30315 cells were transduced with single lentiviruses and selected with 1 ug/mL puromycin for 5 days. Clones of VNPADs were isolated by ring-cloning of surviving colonies and expanded. Two clones (KO1 and KO2, derived from cells transduced with gRNA1 and gRNA2, respectively) were chosen for luciferase assays using the rVSVdG-luc/SARS2 described below. K01 was used for infections with the SARS-CoV-2 Delta variant (described below). To verify knockout, DNA from K01 was purified using QIAGEN DNeasy Blood & Tissue kit (QIAGEN, Germantown, MD, USA, catalog number 69504), and the portion of the *ACE2* gene that was targeted by the gRNA was amplified by PCR (Sequences listed in Supplementary Table S2). PCR products were gel purified and sequenced using Sanger sequencing. Comparisons of sequences were made with the parental cell line to verify knockout of *ACE2* which confirmed premature stop codons in both *ACE2* alleles in clone K01 (Supplementary Figure S2).

### 2.4. Luciferase Assay Using rVSVΔG-luc/SARS2

For luciferase assays, 100,000 SNPADs and VNPADs were seeded in 12-well plates and allowed to become confluent. Once they reached confluency, cells were treated with differentiation media for 10 days as described above. The differentiation media was removed prior to infection. The rVSVΔG-luc/SARS2-S virus (a gift from Melinda Brindley, University of Georgia) has been previously described [28]. We utilized a virus preparation that gave a luciferase calculated amount of 1 × 10^8^ RLUs per µL when 100,000 ACE2-expressing A549 cells were infected for 24 h. The virus was diluted in 1× DMEM + 2% FBS + Pen/Strep and added to the cells, after which they were incubated overnight at 37 °C, 5% CO2. After 24 h, the plates were brought to room temperature and one volume (equal to the amount of media in the well of the plate) of the Nano-Glo^®^ Luciferase Assay Reagent (Promega, Madison, WI, USA, catalog number N1110) was added and gently mixed. One-hundred microliters was transferred to a 96-well white opaque plate for measuring luminescence on a TECAN reader.

### 2.5. Infection of Cells with SARS-CoV-2

Cells were plated in 12-well plates using 100,000 cells/well and allowed to become confluent followed by differentiation for 10 days as described above. Medium was changed to DMEM (Gibco, Grand Island, NY, USA, catalog number 11-965-092) 10% FBS (Gibco, A31605-01) for 1-day. For infection, the media was changed to serum-free media. Each well was infected with virus at an estimated MOI of 1. After 2 h, the cells were washed 3X with PBS (Gibco, 14190-144) and, finally, the PBS was replaced with DMEM 10% FBS. Cells were collected at the indicated time points using TRIZol (ThermoFisher, Waltham, MA, USA, catalog number 15596026).

### 2.6. Reverse Transcriptase Quantitative PCR

Cells in each well of a 12-well plate were lysed using TRIZol followed by RNA extraction as previously described [9]. Reverse transcriptase quantitative polymerase chain reaction (RT-qPCR) was performed as previously described [29] using primers shown in Supplementary Table S1.

### 2.7. Statistical Analyses

GraphPad Prism (version 10.5.0) was used for statistical analysis of the Luciferase Assay and RT-qPCR results. Two-way ANOVA was used to generate statistical differences of *p* < 0.0001 (****), *p* < 0.001 (***), *p* < 0.01 (**), *p* < 0.05 (*). Luciferase and RT-qPCR readings for virus genes were log10 transformed before analysis to account for the unequal variances among each group.

### 2.8. Human Subjects

The study was conducted in accordance with the Declaration of Helsinki, and the protocol for obtaining adipose tissue for isolation of preadipocytes through the University of Iowa Tissue Procurement Core was approved by the University of Iowa Institutional Review Boad (201103721), last approved on 14 December 2024.

## 3. Results

### 3.1. Visceral Adipocytes Express Higher Levels of ACE2 than Subcutaneous Adipocytes

**Figure 1:**
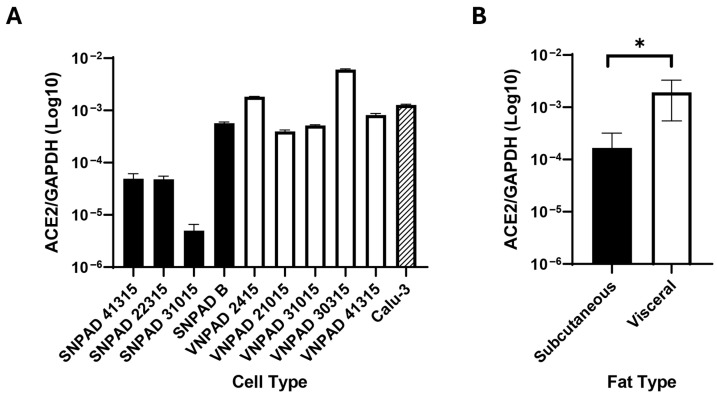
Transcript levels of *ACE2* in differentiated subcutaneous and visceral adipocyte cell lines. (**A**). *ACE2* transcript levels of various differentiated subcutaneous (SNPAD) or visceral (VNPAD) differentiated preadipocyte cell lines. The airway epithelial cell line, Calu3, is shown for comparison. (**B**). Average of *ACE2* transcript levels in the combined differentiated SNPAD and VNPAD cell lines shown in A. Values represent the ratio of *ACE2* over *GAPDH* in log10. An unpaired two-tailed t test was performed on the data for panel (**B**) (* = *p* ≤ 0.05).

Previous studies have indicated that visceral adipose expresses higher levels of ACE2 than subcutaneous adipose and that this correlates with increased levels of SARS-CoV-2 infection [14]. To further study the role of ACE2 in infection of adipocytes, we developed a series of immortalized, subcutaneous nondiabetic preadipocyte (SNPAD) and visceral nondiabetic preadipocyte (VNPAD) cell lines that can be differentiated into adipocytes [23,24,25]. Four subcutaneous SNPAD lines and five visceral VNPAD lines were available for our studies (Supplementary Figure S1). In two cases (numbers 41315 and 31015), subcutaneous and visceral lines were available from the same donor for direct comparison of lines from different depots of the same donor. We assessed the differentiated cells for *ACE2* expression by RT-qPCR and saw significant variability, some with very low and some with relatively high transcript levels compared to Calu-3, an airway epithelial cell line known to be infectable by SARS-CoV-2 (Figure 1A). Levels of *ACE2* trended higher in visceral lines. When the composite measurements of *ACE2* of the different subcutaneous and visceral lines were averaged, there was an overall significant trend of differentiated visceral cell lines having more *ACE2* than differentiated subcutaneous cell lines (Figure 1B, *p* < 0.05), a finding that supports previous studies [14].

### 3.2. Visceral Adipocytes Are More Susceptible to SARS-CoV-2 Spike-Mediated Infection than Subcutaneous Adipocytes

**Figure 2:**
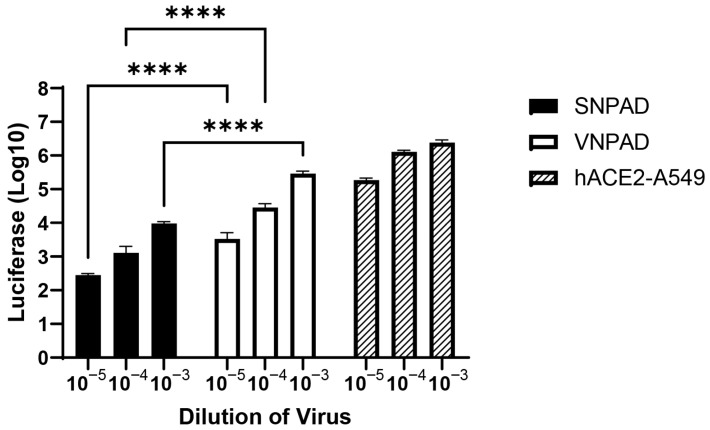
Infection of differentiated subcutaneous and visceral adipocyte cell lines with SARS2-S VSV-luc virus. Values represent luciferase units in log10. Cells were infected with three different dilutions of virus. ACE2-A549 is shown as a positive control. N = 3 biological replicates. A 2-way ANOVA with multiple comparisons was used on log10-transformed data (excluding ACE2-A549) to determine significance (**** = *p* ≤ 0.0001).

For further analysis, we chose to focus on the cell lines VNPAD 30315 D and SNPAD B since they had the highest levels of *ACE2* transcript among the visceral and subcutaneous lines, respectively. To assess SARS-CoV-2 spike (S) mediated susceptibility of the differentiated cells to infection, we used a replication competent VSV-luc in which VSV-G was replaced by SARS-CoV-2 S (original Wuhan variant) [28]. The differentiated visceral VNPAD-30315 D cells had significantly higher levels of infection than differentiated subcutaneous SNPAD B cells (Figure 2), concordant with the various levels of *ACE2* expressed in these cells.

### 3.3. ACE2 Overexpression Increases Susceptibility to SARS-CoV-2 Spike-Mediated Infection

**Figure 3:**
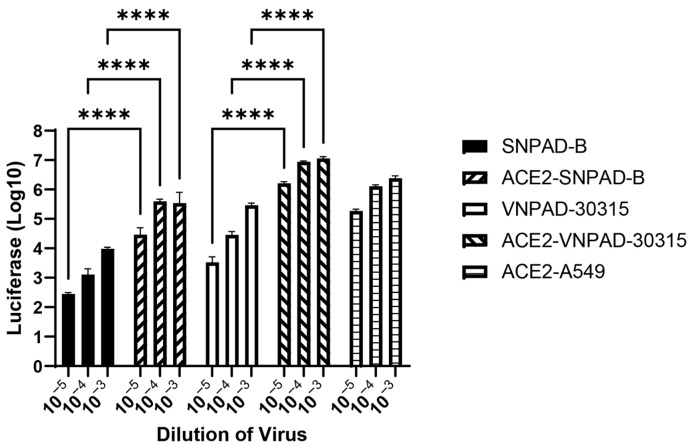
Exogenous ACE2 over-expression (OE) increases infection by SARS2-S VSV-luc virus in subcutaneous and visceral adipocytes. Values represent luciferase units in log10. Preadipocytes were transduced with ACE2, selected, and differentiated before infection with different dilutions of virus. N = 3 biological replicates. A 2-way ANOVA with multiple comparisons was used on log10-transformed data (excluding ACE2-A549) to determine significance (**** = *p* ≤ 0.0001). ACE2-A549 is shown as positive control.

To determine whether increasing ACE2 expression in the subcutaneous and visceral lines would also increase SARS-CoV-2 S-mediated infection, we transduced the undifferentiated subcutaneous SNPAD B and visceral VNPAD 30315 preadipocytes with a retrovirus expressing human ACE2 and selected in puromycin. ACE2 protein expression was low to undetectable by western blot analysis in the parental cell lines, as has been demonstrated previously for adipocytes [21]. However, those cells that were transduced with the ACE2 retrovirus had high expression (Supplementary Figure S1). Overexpression of ACE2 increased susceptibility to SARS-CoV-2 S-mediated infection in the differentiated subcutaneous cells (ACE2-SNPAD-B) bringing levels up to those seen in the parental visceral VNPAD-30315 cells, suggesting that the lower levels of infection in the parental subcutaneous SNPAD B cells as compared to the parental visceral VNPAD-30315 cells are due to lower ACE2 in the subcutaneous cells (Figure 3). Overexpression of ACE2 in the visceral VNPAD-30315 cells also significantly increased susceptibility to infection, indicating that ACE2 is the rate-limiting component to infection (Figure 3).

### 3.4. Knockout of ACE2 Decreases Susceptibility to SARS-CoV-2 Spike-Mediated Infection

**Figure 4:**
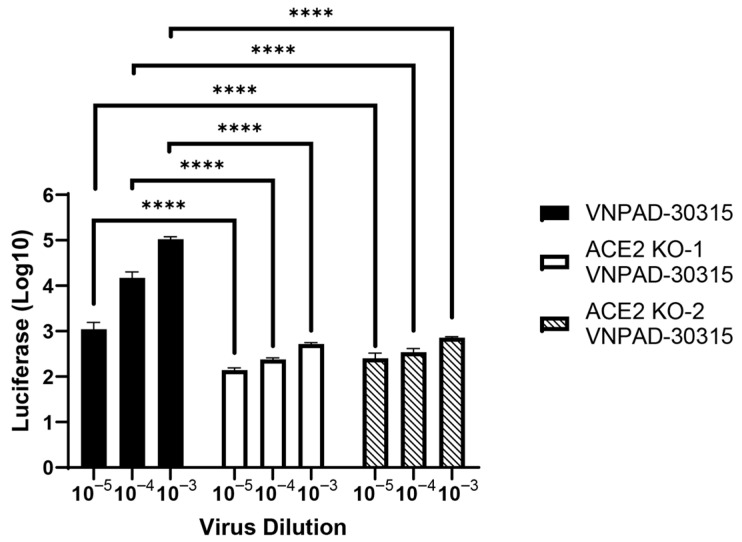
ACE2 knock-out decreases infection by SARS2-S VSV-luc virus. ACE2 was knocked out in visceral preadipocytes using two different guide RNAs (KO1 and KO2) and individual clones were isolated and validated for knockout by sequencing. After differentiation, cells were infected with SARS2-S VSV-luc virus at different concentrations. Values represent luciferase units in log10. N = 3 biological replicates. A 2-way ANOVA with multiple comparisons was used on log10-transformed data to determine significance (**** = *p* ≤ 0.0001).

To determine if loss of ACE2 affected susceptibility to SARS-CoV-2 S-mediated infection, we knocked out ACE2 in the visceral VNPAD-30315 cells with a CAS9-expressing lentiviral vector and two different gRNAs (KO1 and KO2) followed by selection and ring cloning of cells. The resulting cells had significantly decreased susceptibility to SARS-CoV-2 S-mediated infection (Figure 4) compared to the parental visceral cells, again indicating that ACE2 expression is the rate limiting component of infection in these cells.

### 3.5. Dependence on ACE2 for Infection by SARS-CoV-2 Delta Variant

**Figure 5:**
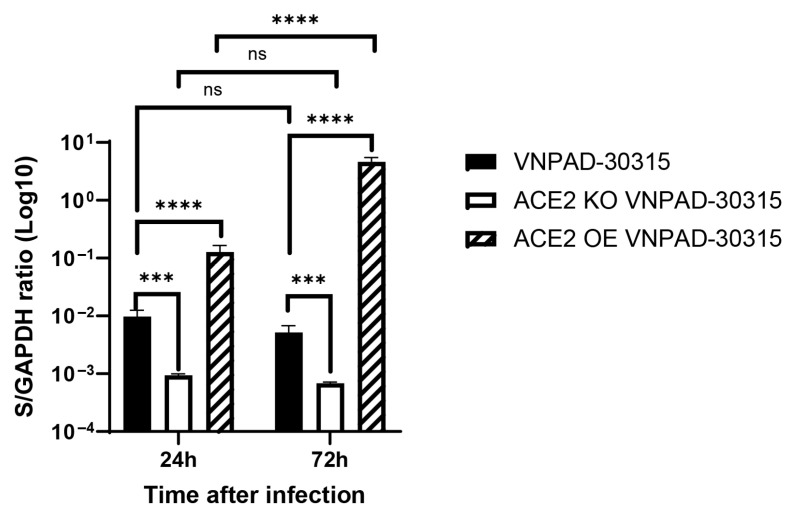
RT-qPCR of the nucleocapsid (*N*) gene in SARS-CoV-2 (Delta)-infected, differentiated VNPAD and ACE2 knock-out (KO) or ACE2 overexpressing (OE) cells with SARS-CoV-2 virus. N = 3 biological replicates. A 2-way ANOVA with multiple comparisons was used on log10-transformed data to determine significance (*** = *p* ≤ 0.001, **** = *p* ≤ 0.0001, ns = nonsignificant).

We then wanted to determine if ACE2 mediated infection by the actual SARS-CoV-2 virus in the differentiated VNPAD-30315 adipocytes. For this purpose, we used the parental visceral VNPAD 30315 cells, an ACE2 knockout clone (KO1 VNPAD-30315) that was verified to have both alleles knocked out (Supplementary Figure S2), and the ACE over-expressing (OE VNPAD-30315) visceral cells. To assess infection, we used an isolate of the SARS-CoV-2 Delta variant in BSL-3 conditions using a single dilution of virus (estimated MOI = 1) or a mock infection. After 6 h, the cells were washed, and media was replaced. Infected cells were collected for RNA after 24 and 72 h followed by RT-qPCR for the S (spike) and N (nucleocapsid) genes of SARS-CoV-2. Virus was detected by RT-qPCR in the parental visceral VNPAD-30315 cells at 24 h but did not show a significant increase at 72 h, suggesting non-productive infection (Figure 5 and Supplementary Figure S3). ACE2 knockout caused a significant decrease in virus detection at 24 and 72 h, supporting the hypothesis that ACE2 mediates infection by the real virus (Figure 5 and Supplementary Figure S3). Similar to what we observed with the S-pseudotyped virus, an increase in viral transcripts was observed with the ACE2 overexpressing (OE) cells as compared to the parental cells at the early timepoint (Figure 5 and Supplementary Figure S3). Interestingly, infected ACE2 OE cells exhibited a significant increase in SARS-CoV-2 gene transcript levels at 72 h as compared to 24 h, suggesting that the cells are permissive for replication but only when enough virus enters into these cells through ACE2-mediated infection.

### 3.6. Proinflammatory Response in SARS-CoV-2 Infected Cells

**Figure 6:**
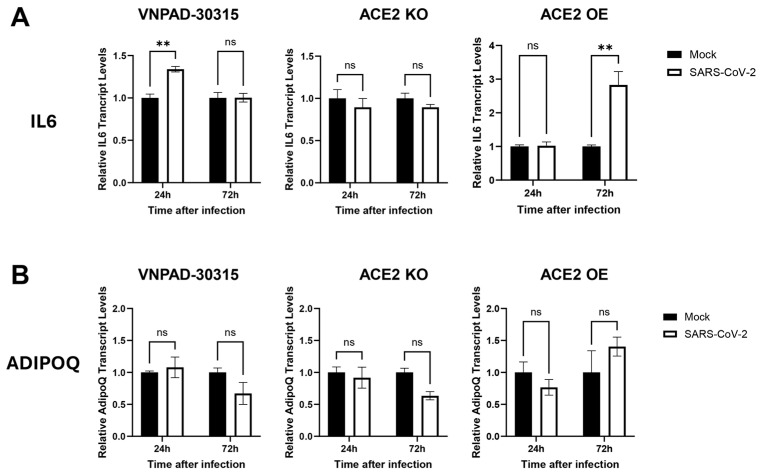
RT-qPCR of *IL6* and *ADIPOQ* in SARS-CoV-2 (Delta)-infected, differentiated VNPAD and ACE2 knock-out (KO) or ACE2 overexpressing (OE) cells with SARS-CoV-2 virus. (**A**). *IL6* relative transcript levels in the different conditions (mock versus infected) and timepoints (24 h and 72 h) post infection, (**B**). *ADIPOQ* relative transcript levels in the same conditions and at the same timepoints as in (**A**). Values are relative to mock infection at that particular time point. A 2-way ANOVA with multiple comparisons was used to determine significance (** = *p* ≤ 0.01, ns = nonsignificant).

Finally, we assessed levels of *IL6* and adiponectin (*ADIPOQ*) in the SARS-CoV-2 infected cells as surrogates for induction of a proinflammatory response and adipocyte differentiation, respectively. Increased IL6 is associated with increased mortality in COVID-19 patients [30]. Infection of the parental visceral VNPAD-30315 cells caused a low level of increase in *IL6* transcripts but only at 24 h, not at 72 h. (Figure 6A). ACE2 KO cells had no increase in *IL6* levels at 24 or 72 h. The ACE2 OE cells exhibited no increase in *IL6* transcript levels at 24 h and a modest increase at 72 h (Figure 6A), indicating that more virus in these cells is associated with an increased proinflammatory response. Transcript levels of *ADIPOQ* remained unchanged as compared to mock-infected cells in any condition (Figure 6B), suggesting that infection did not significantly alter adipocyte function at these time points.

## 4. Discussion

### 4.1. The Role of ACE2 and Other Factors in SARS-CoV-2 Infection of Adipocytes

The role of SARS-CoV-2 infection of cells other than airway epithelial cells in pathogenesis continues to be a matter of interest and debate. Many cell types have been implicated in being infected by the virus, and that infection has been proposed to be involved in causing disease, associated with acute and chronic (e.g., long COVID) pathology. SARS-CoV-2 infection of adipocytes has attracted attention because the involvement of adipose tissue may help explain why increased adiposity, particularly visceral adiposity, is such a strong co-morbidity in COVID. Previous studies have demonstrated that adipocytes are susceptible to SARS-CoV-2 infection [14,19,21,30,31,32]. However, different studies have indicated either involvement or no involvement of the canonical mechanism of entry through the ACE2 receptor. We showed here that visceral adipocytes express higher levels of ACE2 and are more susceptible to SARS-CoV-2 S-mediated infection than subcutaneous adipocytes. Furthermore, we demonstrate that overexpression of ACE2 increases susceptibility and knockout of ACE2 decreases susceptibility to infection of visceral adipocytes.

Different studies, using transcriptomic data, have shown that adipose tissue has relatively high *ACE2* transcript levels compared to other tissues [33] whereas protein expression in adipose tissue was found to be low [34]. While we were able to detect relatively high levels of *ACE2* transcript in differentiated adipocytes in vitro, protein levels were undetectable by western blot analysis unless ACE2 was exogenously overexpressed (Supplementary Figure S1). Low or undetectable levels of ACE2 protein in visceral adipocytes as assessed by western blot analysis has been reported by another group [21]. Why this is the case is unclear, but it is possible that, while ACE2 protein is not expressed at high levels in adipocytes, the small levels that are expressed play a role in entry, as our studies indicate. While our studies provide evidence that ACE2 is important for infection of visceral adipocytes, we cannot rule out that other factors are needed for infection. For example, we saw increased transcript levels of the protease gene *TMPRRS2* in visceral adipocytes as compared to subcutaneous adipocytes (Supplementary Figure S4). It should be noted, however, that adipose tissue expresses low levels of TMPRSS2 as compared to other tissues [35], so it is unclear if TMPRRS2 is important for adipocyte infection. Further, it is possible that the infection of adipocytes involves mechanisms in addition to ACE2 and TMPRRS2. The virus can enter cells through cathepsin-mediated endocytosis without TMPRSS2 involvement [36], and there is evidence that infection can be mediated through other factors such as GRP78, a binding partner to ACE2 [37,38] and phosphatidylserine receptor mechanisms [39]. Indeed, GRP78 is highly expressed in visceral adipose tissue, particularly in association with increased age, obesity, and diabetes [38]. Further studies will be needed to address the role of these and other factors in SARS-CoV-2 entry into adipocytes and their potential involvement in pathogenesis.

### 4.2. Permissivity of Adipocytes to SARS-CoV-2 Replication

The consequences of SARS-CoV-2 entry into adipocytes with regard to replication and viral persistence are not completely clear. In our studies, an increase in viral transcript levels at a later time point was only observed in cells that overexpressed ACE2, suggesting that adipocytes with higher ACE2 are permissive for infection. Results from other groups have suggested permissivity as well [14,20]. Our results suggest that visceral adipocytes at baseline are susceptible to infection but not very permissive for replication. However, high ACE2 expression, due to exogenous expression or other mechanisms, and the concomitant higher levels of SARS-CoV-2 entry might confer increased permissivity because of increased evasion of the innate immune response in the cells or other factors. ACE2 expression in adipose tissue is regulated by diet and obesity [40,41,42], and it is likely that variable levels of ACE2 expression in adipocyte populations occur naturally. This could lead to differential levels of susceptibility and permissivity in these cells which, in turn, could play a role in viral persistence and severity of disease.

### 4.3. Proinflammatory Responses Caused by SARS-CoV-2 Infection of Adipocytes

In our studies, we saw evidence of a proinflammatory response caused by SARS-CoV-2 infection of visceral adipocytes, as indicated by higher levels of *IL6* transcript, but only in those cells that overexpressed ACE2 and then only at a later time point of 72 h. A proinflammatory response has also been observed upon infection of adipocytes in other studies [14,20,21,31]. Our results suggest that adipocytes with high ACE2 expression may be more susceptible to a proinflammatory response caused by infection. Increased inflammation caused by adipose infection could potentially be related to why visceral obesity increases the risk of COVID-associated severe disease and mortality [30]. Whether this is due to adipose or adipocyte infection specifically is unclear. Adipose inflammation is an important early event in the development of metabolic syndrome, including insulin resistance. Patients who have had severe COVID are at increased risk for diabetes [8,43], but it is unknown if this is related to adipose inflammation caused by COVID. While our studies indicated a proinflammatory response caused by SARS-CoV-2 infection of ACE2-expressing adipocytes, we did not observe a significant change in transcript levels of adiponectin at the timepoints evaluated, suggesting little change in adipocyte differentiation and function. However, it is possible that assessment of expression of other proinflammatory and differentiation-associated genes or analysis at later time points would show an effect. Further studies are needed to address what proinflammatory and adipocyte-specific factors are affected by infection of adipose and adipocytes, specifically, and the consequences that this might have on severity or long-term effects of COVID.

## Data Availability

Upon publication, all relevant research data will be made publicly available on Iowa Research Online.

## Supplementary Materials

The following supporting information can be downloaded at: https://www.mdpi.com/article/10.3390/v17101311/s1, Supplemental Table S1: Human Preadipocyte Cell lines; Supplemental Table S2: Primary and gRNAs; Supplemental Figure S1: Western blot analysis of ACE2 in parental and ACE2 over-expression cells; Supplemental Figure S2: Frameshifts in both alleles of VNPAD 30315 ACE2 knockout (KO) cells leads to premature stop codons; Supplemental Figure S3: RT-qPCR of the *S* gene in SARS-CoV-2 (Delta)-infected, differentiated VNPAD and ACE2 knock-out (KO) or ACE2 overexpressing (OE) cells; Supplemental Figure S4: TMPRSS2 in differentiated SNPAD B and VNPAD 30315 cells compared to Calu3.

## Abbreviations

The following abbreviations are used in this manuscript:

SARS-CoV-2	Severe Acute Respiratory Syndrome Coronavirus 2
COVID-19	Coronavirus Disease 19
ACE2	Angiotensin Converting Enzyme 2
TMPRRS2	Transmembrane Serine Protease 2
IL6	Interleukin 6
VSV	Vesicular Stomatitis Virus
S	Spike
N	Nucleocapsid
RT-qPCR	Reverse Transcriptase quantitative Polymerase Chain Reaction
OE	Overexpression
KO	Knockout
ANOVA	Analysis of Variance
VNPAD	Visceral normal preadipocyte
SNPAD	Subcutaneous normal preadipocytes

## References

[1] Best J.H., Mohan S.V., Kong A.M., Patel K., Pagel J.M., Ivanov B., Brawley O.W., Jariwala-Parikh K., Zazzali J.L., Pauk J. (2020). Baseline Demographics and Clinical Characteristics Among 3471 US Patients Hospitalized with COVID-19 and Pulmonary Involvement: A Retrospective Study. Adv. Ther..

[2] Hamer M., Gale C.R., Kivimaki M., Batty G.D. (2020). Overweight, obesity, and risk of hospitalization for COVID-19: A community-based cohort study of adults in the United Kingdom. Proc. Natl. Acad. Sci. USA.

[3] Kruglikov I.L., Scherer P.E. (2020). The Role of Adipocytes and Adipocyte-Like Cells in the Severity of COVID-19 Infections. Obesity (Silver Spring).

[4] Kruglikov I.L., Shah M., Scherer P.E. (2020). Obesity and diabetes as comorbidities for COVID-19: Underlying mechanisms and the role of viral-bacterial interactions. Elife.

[5] Ryan P.M., Caplice N.M. (2020). Is Adipose Tissue a Reservoir for Viral Spread, Immune Activation, and Cytokine Amplification in Coronavirus Disease 2019?. Obesity (Silver Spring).

[6] Rosen E.D., Spiegelman B.M. (2014). What we talk about when we talk about fat. Cell.

[7] Cohen P., Spiegelman B.M. (2016). Cell biology of fat storage. Mol. Biol. Cell.

[8] Montefusco L., Ben Nasr M., D’Addio F., Loretelli C., Rossi A., Pastore I., Daniele G., Abdelsalam A., Maestroni A., Dell’Acqua M. (2021). Acute and long-term disruption of glycometabolic control after SARS-CoV-2 infection. Nat. Metab..

[9] Gourronc F.A., Rebagliati M.R., Kramer-Riesberg B., Fleck A.M., Patten J.J., Geohegan-Barek K., Messingham K.N., Davey R.A., Maury W., Klingelhutz A.J. (2022). Adipocytes are susceptible to Ebola Virus infection. Virology.

[10] Mery G., Epaulard O., Borel A.L., Toussaint B., Le Gouellec A. (2020). COVID-19: Underlying Adipokine Storm and Angiotensin 1-7 Umbrella. Front. Immunol..

[11] Favre G., Legueult K., Pradier C., Raffaelli C., Ichai C., Iannelli A., Redheuil A., Lucidarme O., Esnault V. (2020). Visceral fat is associated to the severity of COVID-19. Metabolism.

[12] Foldi M., Farkas N., Kiss S., Dembrovszky F., Szakacs Z., Balasko M., Eross B., Hegyi P., Szentesi A. (2020). Visceral adiposity elevates the risk of critical condition in COVID-19: A systematic review and meta-analysis. Obesity (Silver Spring).

[13] Han T., Kang J., Li G., Ge J., Gu J. (2020). Analysis of 2019-nCoV receptor ACE2 expression in different tissues and its significance study. Ann. Transl. Med..

[14] Saccon T.D., Mousovich-Neto F., Ludwig R.G., Carregari V.C., Dos Anjos Souza A.B., Dos Passos A.S.C., Martini M.C., Barbosa P.P., de Souza G.F., Muraro S.P. (2022). SARS-CoV-2 infects adipose tissue in a fat depot- and viral lineage-dependent manner. Nat. Commun..

[15] Gaussen A., Hornby L., Rockl G., O’Brien S., Delage G., Sapir-Pichhadze R., Drews S.J., Weiss M.J., Lewin A. (2021). Evidence of SARS-CoV-2 Infection in Cells, Tissues and Organs and the Risk of Transmission Through Transplantation. Transplantation.

[16] Varga Z., Flammer A.J., Steiger P., Haberecker M., Andermatt R., Zinkernagel A.S., Mehra M.R., Schuepbach R.A., Ruschitzka F., Moch H. (2020). Endothelial cell infection and endotheliitis in COVID-19. Lancet.

[17] Puelles V.G., Lutgehetmann M., Lindenmeyer M.T., Sperhake J.P., Wong M.N., Allweiss L., Chilla S., Heinemann A., Wanner N., Liu S. (2020). Multiorgan and Renal Tropism of SARS-CoV-2. N. Engl. J. Med..

[18] Zickler M., Stanelle-Bertram S., Ehret S., Heinrich F., Lange P., Schaumburg B., Kouassi N.M., Beck S., Jaeckstein M.Y., Mann O. (2022). Replication of SARS-CoV-2 in adipose tissue determines organ and systemic lipid metabolism in hamsters and humans. Cell Metab..

[19] Basolo A., Poma A.M., Bonuccelli D., Proietti A., Macerola E., Ugolini C., Torregrossa L., Giannini R., Vignali P., Basolo F. (2022). Adipose tissue in COVID-19: Detection of SARS-CoV-2 in adipocytes and activation of the interferon-alpha response. J. Endocrinol. Investig..

[20] Reiterer M., Rajan M., Gomez-Banoy N., Lau J.D., Gomez-Escobar L.G., Ma L., Gilani A., Alvarez-Mulett S., Sholle E.T., Chandar V. (2021). Hyperglycemia in acute COVID-19 is characterized by insulin resistance and adipose tissue infectivity by SARS-CoV-2. Cell Metab..

[21] Martinez-Colon G.J., Ratnasiri K., Chen H., Jiang S., Zanley E., Rustagi A., Verma R., Chen H., Andrews J.R., Mertz K.D. (2022). SARS-CoV-2 infection drives an inflammatory response in human adipose tissue through infection of adipocytes and macrophages. Sci. Transl. Med..

[22] Steenblock C., Bechmann N., Beuschlein F., Wolfrum C., Bornstein S.R. (2023). Do adipocytes serve as a reservoir for severe acute respiratory symptom coronavirus-2?. J. Endocrinol..

[23] Vu B.G., Gourronc F.A., Bernlohr D.A., Schlievert P.M., Klingelhutz A.J. (2013). Staphylococcal superantigens stimulate immortalized human adipocytes to produce chemokines. PLoS ONE.

[24] Gourronc F.A., Robertson L.W., Klingelhutz A.J. (2018). A delayed proinflammatory response of human preadipocytes to PCB126 is dependent on the aryl hydrocarbon receptor. Environ. Sci. Pollut. Res. Int..

[25] Murphy A.R., Asif H., Cingoz H., Gourronc F.A., Ankrum J.A., Klingelhutz A.J., Kim J.J. (2024). The Impact of High Adiposity on Endometrial Progesterone Response and Metallothionein Regulation. J. Clin. Endocrinol. Metab..

[26] Gadupudi G., Gourronc F.A., Ludewig G., Robertson L.W., Klingelhutz A.J. (2015). PCB126 inhibits adipogenesis of human preadipocytes. Toxicol. In Vitro.

[27] Shalem O., Sanjana N.E., Hartenian E., Shi X., Scott D.A., Mikkelson T., Heckl D., Ebert B.L., Root D.E., Doench J.G. (2014). Genome-scale CRISPR-Cas9 knockout screening in human cells. Science.

[28] Havranek K.E., Jimenez A.R., Acciani M.D., Lay Mendoza M.F., Reyes Ballista J.M., Diaz D.A., Brindley M.A. (2020). SARS-CoV-2 Spike Alterations Enhance Pseudoparticle Titers and Replication-Competent VSV-SARS-CoV-2 Virus. Viruses.

[29] Gourronc F.A., Perdew G.H., Robertson L.W., Klingelhutz A.J. (2020). PCB126 blocks the thermogenic beiging response of adipocytes. Environ. Sci. Pollut. Res. Int..

[30] Currey J., Ellsworth C., Khatun M.S., Wang C., Chen Z., Liu S., Midkiff C., Xiao M., Ren M., Liu F. (2024). Upregulation of inflammatory genes and pathways links obesity to severe COVID-19. Biochim. Biophys. Acta Mol. Basis Dis..

[31] Quaranta P., Scabia G., Storti B., Dattilo A., Quintino L., Perrera P., Di Primio C., Costa M., Pistello M., Bizzarri R. (2024). SARS-CoV-2 Infection Alters the Phenotype and Gene Expression of Adipocytes. Int. J. Mol. Sci..

[32] Thangavel H., Dhanyalayam D., Lizardo K., Oswal N., Dolgov E., Perlin D.S., Nagajyothi J.F. (2023). Susceptibility of Fat Tissue to SARS-CoV-2 Infection in Female hACE2 Mouse Model. Int. J. Mol. Sci..

[33] Li M.Y., Li L., Zhang Y., Wang X.S. (2020). Expression of the SARS-CoV-2 cell receptor gene ACE2 in a wide variety of human tissues. Infect. Dis. Poverty.

[34] Hikmet F., Mear L., Edvinsson A., Micke P., Uhlen M., Lindskog C. (2020). The protein expression profile of ACE2 in human tissues. Mol. Syst. Biol..

[35] Cao W., Feng Q., Wang X. (2021). Computational analysis of TMPRSS2 expression in normal and SARS-CoV-2-infected human tissues. Chem. Biol. Interact..

[36] Pislar A., Mitrovic A., Sabotic J., Pecar Fonovic U., Perisic Nanut M., Jakos T., Senjor E., Kos J. (2020). The role of cysteine peptidases in coronavirus cell entry and replication: The therapeutic potential of cathepsin inhibitors. PLoS Pathog..

[37] Carlos A.J., Ha D.P., Yeh D.W., Van Krieken R., Tseng C.C., Zhang P., Gill P., Machida K., Lee A.S. (2021). The chaperone GRP78 is a host auxiliary factor for SARS-CoV-2 and GRP78 depleting antibody blocks viral entry and infection. J. Biol. Chem..

[38] Shin J., Toyoda S., Nishitani S., Fukuhara A., Kita S., Otsuki M., Shimomura I. (2021). Possible Involvement of Adipose Tissue in Patients With Older Age, Obesity, and Diabetes With SARS-CoV-2 Infection (COVID-19) via GRP78 (BIP/HSPA5): Significance of Hyperinsulinemia Management in COVID-19. Diabetes.

[39] Bohan D., Van Ert H., Ruggio N., Rogers K.J., Badreddine M., Aguilar Briseno J.A., Elliff J.M., Rojas Chavez R.A., Gao B., Stokowy T. (2021). Phosphatidylserine receptors enhance SARS-CoV-2 infection. PLoS Pathog..

[40] Gupte M., Boustany-Kari C.M., Bharadwaj K., Police S., Thatcher S., Gong M.C., English V.L., Cassis L.A. (2008). ACE2 is expressed in mouse adipocytes and regulated by a high-fat diet. Am. J. Physiol. Regul. Integr. Comp. Physiol..

[41] Gomez-Zorita S., Milton-Laskibar I., Garcia-Arellano L., Gonzalez M., Portillo M.P. (2021). An Overview of Adipose Tissue ACE2 Modulation by Diet and Obesity. Potential Implications in COVID-19 Infection and Severity. Int. J. Mol. Sci..

[42] Salazar M., Ferreira M., Oliveira S.M., Saraiva F., Pinho C., Jarnalo M., Correia-Sa I., Falcao-Pires I., Leite-Moreira A., Neves D. (2025). Impact of Obesity and Ageing on the Expression of Key Mediators of SARS-CoV-2 Infection in Human Adipose Tissue. Int. J. Mol. Sci..

[43] Kim S.H., Arora I., Hsia D.S., Knowler W.C., LeBlanc E., Mylonakis E., Pratley R., Pittas A.G. (2023). New-Onset Diabetes After COVID-19. J. Clin. Endocrinol. Metab..

