# Supplementary material for: ACE2-Mediated Infection of Immortalized Human Visceral Adipocytes by SARS-CoV-2
Source: Viruses. 2025 Sep 27;17(10):1311. doi: 10.3390/v17101311 (PMC12567992; doi:10.3390/v17101311)
Supplement: Supplementary file 1 [file viruses-17-01311-s001.zip › viruses-3831983-supplementary.pdf]

Supplemental Table S1: Human Preadipocyte Cell Lines

Cell Line	Depot Derivation	Age	Sex	Reference
SNPAD 41315[c]	Subcutaneous	61	F	none
SNPAD 22315	Subcutaneous	52	M	none
SNPAD 31015[b]	Subcutaneous	23	F	none
(S)NPAD B[a,d]	Subcutaneous	38	F	(Vu et al., 2013)[d]
VNPAD 2415	Visceral	49	F	(Murphy et al., 2024)
VNPAD 21015	Visceral	38	F	none
VNPAD 31015[b]	Visceral	23	F	none
VNPAD 30315 D[a]	Visceral	51	F	(Murphy et al., 2024)
VNPAD 41315[c]	Visceral	61	F	none

[a]Clone

[b]Same donor 31015

[c]Same donor 41315

[d]Subcutaneous line referred to as NPAD B in previous publications

Supplemental Table S2: Primers and gRNAs

Primer Name	Sequence (All primers are 5’ to 3’)
ACE2 forward	ACTTGGACCTCCTAACCAGC
ACE2 reverse	CGATGGAGGCATAAGGATTTTCT
GAPDH forward	AAGGTCATCCATGACAACTTTG
GAPDH reverse	GTAGAGGCAGGGATGATGTTCT
SARS-Cov-2 N forward	ACCCGCAATCCTGCTAACAA
SARS-Cov-2 N reverse	ACGAGAAGAGGCTTGACTGC
SARS-Cov-2 S forward	AACGCCACCAGATTTGCATC
SARS-Cov-2 S reverse	GTTTGCCCTGGAGCGATTTG
TMPRRS2 forward	TGGAAGTTCATGGGCAGCAA
TMPRRS2 reverse	GTTTGGTCCGTAGAGGCGAA
IL6 forward	CAATCTGGATTCAATGAGGAGAC
IL6 reverse	CTCTGGCTTGTTCCTCACTACTC
ADIPOQ forward	GCTCAGCAT TCA GTG TGG GA
ADIPOQ reverse	GTA CAG CCC AGG AAT GTT GC
ACE2 gRNA #1	ACAGTTTAGACTACAATGAGAGG
ACE2 gRNA #2	CCAAAGGCGAGAGATAGTTGGGG
Genomic ACE2 PCR forward (F1)	GCTTATTACTTGAACCAGGTAGGC
Genomic ACE2 PCR reverse (R1)	TCAAAGGGCAGGCTTGGTAA
ACE2 primer for sequencing forward gRNA#1	TGATTGCAGATATGTGTGTTTCAA
ACE2 primer for sequencing reverse gRNA#1	TGCAGAAGAAATAGCCCCGT

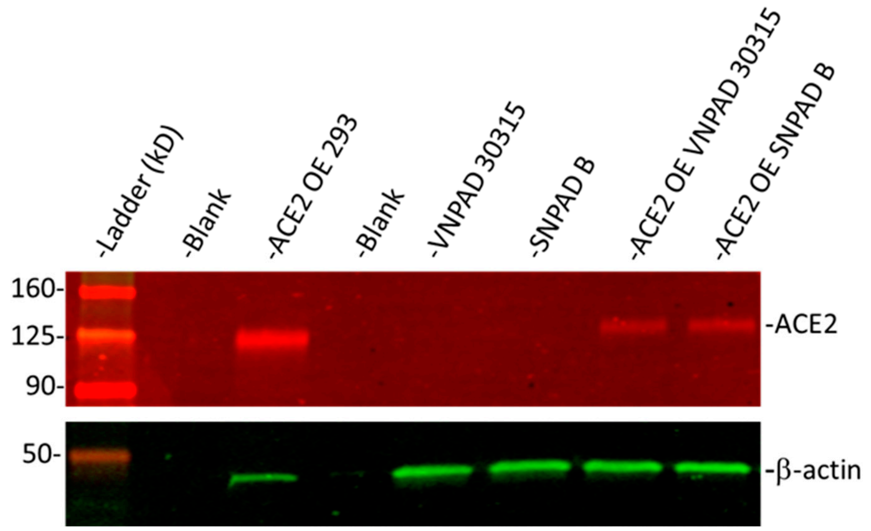

Supplemental Figure S1: Western analysis of ACE2 in parental and ACE2 over-expression (OE) cells. Cells were transduced with human ACE2 as described in the Materials and Methods. Differentiated adipocytes were collected for protein and levels of ACE2 were assessed by western analysis. An antibody for actin was used as a loading control.

gRNA#1		AC	AGT	TTA	GAC	TAC	AAT	GAG	AGG											
Wildtype	GCA	AAC	AGT	TTA	GAC	TAC	AAT	GAG	AGG	CTC	TGG	GCT	TGG	GAA	AGC	TGG	AGA	TCT	GAG	...
	A	N	S	L	D	Y	N	E	R	L	W	A	W	E	S	W	R	S	E	
Allele 1	GCA	AAC	AGT	TTA	GAC	TAC	AAT	TGA	GAG	GCT	CTG	GGC	TTG	GGA	AAG	CTG	GAG	ATC	TGA	...
	A	N	S	L	D	Y	N	**Stop**	E	A	L	G	L	G	L	L	E	I	**Stop**	
Allele 2	GCA	AAC	AGT	CAA	CAG	TGC	AAA	CGA	GAG	GCT	CTG	GGC	TTG	GGA	AAG	CTG	GAG	ATC	TGA	...
	A	N	S	Q	Q	C	L	R	E	A	L	G	L	G	L	L	E	I	**Stop**	

Supplemental Figure S2: Frameshifts in both alleles of VNPAD 30315 ACE2 knockout (KO) cells leads to premature stop codons. DNA was extracted from cells and ACE2 was amplified and sequenced as described in the Materials and Methods. Red indicates nucleotides inserted in region of gRNA by CRISPR/Cas. Blue indicates stop codon caused by frameshift.

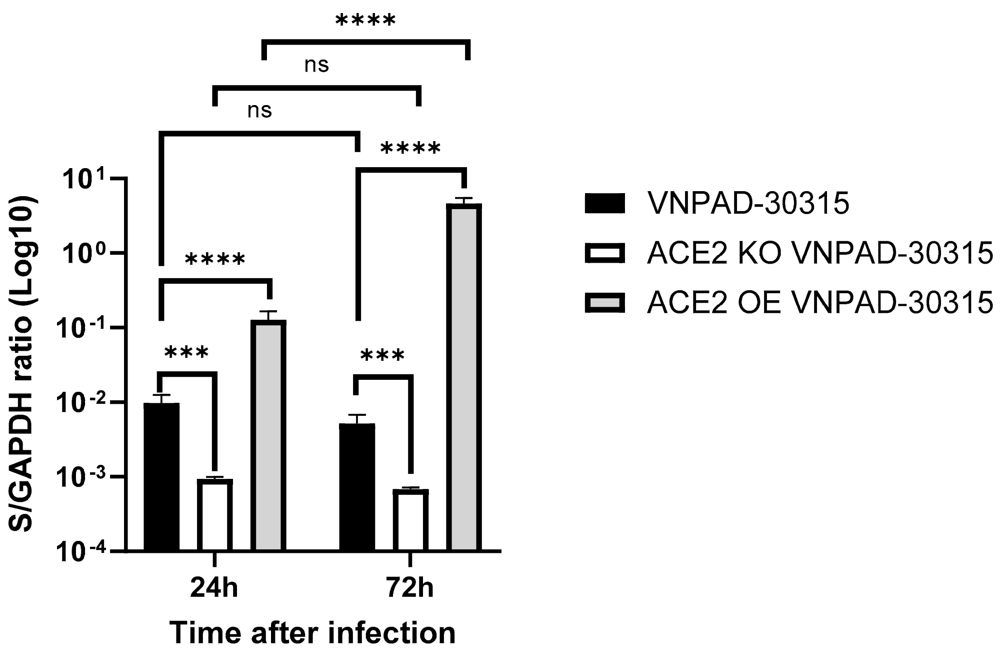

Supplemental Figure S3: RT-qPCR of the *S* gene in SARS-CoV-2 (Delta)-infected, differentiated VNPAD and *ACE2* knock-out (KO) or ACE2 overexpressing (OE) cells. N=3 biological replicates. A 2-way ANOVA with multiple comparisons was used on log10-transformed data to determine significance.

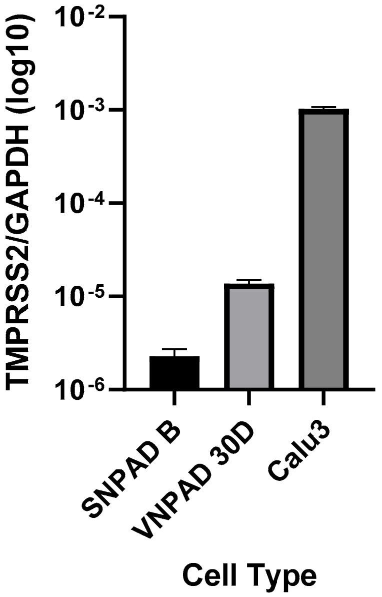

Supplemental Figure S4: *TMPRSS2* in differentiated SNPAD B and VNPAD 30315 cells compared to Calu3. Levels of *TMPRSS2* and *GAPDH* were assessed by RT-qPCR as described in the Materials and Methods.
